# Center of Mass Feedback for joint torque control: a generalizable approach for balance augmentation in wearable robots

**DOI:** 10.21203/rs.3.rs-8605659/v1

**Published:** 2026-02-13

**Authors:** Kristen L. Jakubowski, Gregory S. Sawicki, Lena H. Ting

**Affiliations:** Emory University and Georgia Institute of Technology; Georgia Institute of Technology; Emory University and Georgia Institute of Technology

**Keywords:** sensorimotor feedback, feedforward control, postural control

## Abstract

**Background:**

Exoskeletons have the potential to augment balance and decrease fall risk. However, existing balance-augmenting wearable robot controllers have only been tested in single planes of motion during either standing or walking. Thus, it is unclear whether a single control scheme can generalize across perturbations with varying spatial properties or from standing to walking. Inspired by the nervous system’s generalizable balance control strategy across perturbation types and conditions, we propose a novel torque control framework that modulates multi-joint reactive torques based on center of mass (CoM) deviation. We evaluated the generalizability of our delayed CoM feedback controller to predict multi-joint torque responses to perturbations of varying magnitudes, directions, and across movement contexts.

**Methods:**

In nine healthy young adults, we tested the ability of a delayed CoM feedback scheme to predict multi-joint torque responses to 1) ramp-and-hold support surface perturbations at three magnitudes in 8 directions, 2) a continuous sinusoidal movement, mimicking cyclical tasks like walking, and 3) a sinusoidal motion with random perturbations superimposed to mimic perturbations during cyclic tasks. We trained the model on single ramp-and-hold conditions and evaluated its ability to generalize to all others.

**Results:**

The delayed CoM feedback controller trained on a single ramp-and-hold condition generalized to all ramp-and-hold perturbations for all joints, predicting the joint torques for perturbations of varying directions and magnitudes with high fidelity (average R^2^ > 0.84 and RMSE < 0.08 Nm/kg). However, generalization from standing to cyclic movement only occurred for hip and knee flexion. The CoM feedback parameters from ramp-and-hold perturbations generalized to the continuous sinusoidal movement (cyclic movement) and the sinusoidal movement with superimposed perturbations (unexpected perturbations) for hip flexion and knee flexion (average R^2^>0.70 and RMSE < 0.13 Nm/kg), but not for ankle plantarflexion and hip adduction (R^2^>0.20 and RMSE < 0.22 Nm/kg).

**Conclusion:**

Our findings show that a physiologically-inspired CoM feedback controller can robustly predict balance-correcting torques appropriate for driving a hip or knee wearable robotic device during standing and movement, and an ankle device during standing only. The goodness-of-fit to joint torque is comparable to top machine learning algorithms, yet requires orders of magnitude less training data, enabling rapid implementation to reduce fall risk.

## Background

Over the past few decades, there has been significant development of exoskeletons for enhancing human mobility [1–3]. These exoskeletons have primarily focused on reducing energy expenditure during steady-state behaviors [4–6], despite the desire from both users and clinicians for devices that can aid in balance [7, 8]. Given this demand, there has been a recent push to develop exoskeletons as a balance-augmenting tool [9–15]. In this emerging field, several challenges remain open that limit the current impact of balance-augmenting exoskeletons. First, current balance-augmenting exoskeletons have not been evaluated in perturbations with varying spatial and temporal features. Perturbations that vary in direction, magnitude, and timing will challenge balance in very different ways [16, 17]. Second, current balance-augmenting exoskeletons actuate either the ankle [9–13] or the hip [14, 15], and do not account for coordinated multi-joint responses. Humans maintain balance in response to a postural perturbation by coordinating the reactive torques across the hip, knee, and ankle, with the engagement of each joint varying depending on the spatiotemporal features of the perturbation and the individual’s overall response strategy [18–20]. Lastly, the exoskeleton control scheme should be robust to perturbations during both standing and walking, as falls occur during both [21]. As such, the ideal control scheme for balance augmentation for a wearable robotic device (exoskeleton, exosuit, or prosthesis) would be robust and generalizable to perturbations with varying spatiotemporal features, work during both standing and walking, and be adaptable to variations in the human physiological response.

Although exoskeleton controllers based on perturbation characteristics have been effective at augmenting balance, they may have limited generalizability and accuracy for real-time balance augmentation. Recently, balance augmentation has been achieved in ankle exoskeletons that detect external perturbations [11, 12], but it is unclear how this control strategy could be implemented to account for a coordinated multi-joint response. Further, the generalizability of perturbation-based approaches has not been evaluated, as they have only been tested against a single direction of perturbation. Lastly, accurate estimation of the external disturbance is required, which is currently achieved through high-fidelity laboratory-based measures and exact knowledge of the applied perturbation [11]. While this could theoretically be achieved using wearable sensors, it has yet to be experimentally validated. Additionally, relying on wearable sensors for providing perturbation information may limit ecological relevance and balance-augmenting performance. The fastest a perturbation can currently be detected is approximately 150 ms [10], but acting faster than the physiological response (~ 50ms after perturbation onset), improves balance compared to actuating with the physiological muscle response (~ 150ms after perturbation onset) [12]. As such, relying on wearable sensor data to provide information on the applied perturbation for actuation may result in the exoskeleton actuating too late to augment balance.

Basing exoskeleton control on the human physiological balance response may be a promising approach for achieving generalizability while also capturing variation in responses across perturbation spatial properties and contexts. CoM state is related to the balance correcting response during both standing [22–25] and walking [26–28]. Specifically, we have previously demonstrated that a delayed CoM feedback model can accurately predict the muscle activation patterns elicited in multiple leg muscles in response to perturbations of varying direction and amplitude during standing balance [22–25]. Such a control strategy explains the inter-joint coupling across perturbations varying in complexity and direction, resulting in a single control signal (CoM deviation) that robustly drives the electromyography (EMG) response across multiple joints [22–25]. While this control strategy can explain the muscle activation response, controlling an exoskeleton requires commanding torques, and it is not trivial to transition from the muscle activation response to a joint torque response, especially at multiple joints. Thus, we recently extended this principle to show that delayed CoM feedback also underlies the multi-joint torque response to perturbations of varying magnitude during standing [20]. Moreover, CoM state (position or velocity) control has been successfully implemented into ankle exoskeletons for balance augmentation during both standing and walking [9, 10, 13]. However, CoM state control schemes have only been tested in the anterior-posterior direction, during either standing or walking, not both, and only at the ankle joint. Lastly, this method eliminates the need for a perturbation detector. The joint torque control signal to the exoskeleton is continuously updated based on CoM deviation. Thus, the only limitation to actuating faster than the physiological muscle activation response, which is needed to augment balance [12], would be the bandwidth of the device. Collectively, while this approach seems promising, it remains to be seen whether it works at joints other than the ankle and generalizes across perturbations with varying spatiotemporal properties and movement contexts (e.g., standing and walking).

This study aimed to evaluate whether a delayed CoM feedback control scheme for lower limb wearable robotic devices can generalize across perturbations of varying magnitudes and directions and across different movement contexts. We evaluate the ability of the CoM feedback model to predict the response to: 1) ramp-and-hold perturbations of varying direction within the same magnitude, 2) Ramp-and-hold perturbations of varying magnitude within the same direction, 3) Sinusoidal support surface movements within the same direction to mimic walking, and 4) Sinusoidal support surface movements with superimposed perturbations to mimic perturbations during walking. We used our previously developed CoM feedback model [20] to predict the biological torque response in ankle plantarflexion/dorsiflexion, knee flexion/extension, hip flexion/extension, and hip ab/adduction. We expected that the CoM feedback model would be able to generalize across conditions and movement contexts—performance that would support the future implementation of this approach within an exoskeleton, exosuit, lower-limb prosthesis, or humanoid robots to augment balance. A small subset of this data has previously been published [29].

## Materials and Methods

### Participants

Nine healthy young adults (5 females and 4 males; age 22 ± 4 years; height 1.72 ± 0.08 m; mass 69 ± 11 kg) provided informed consent to participate in this study. All participants were free of neurological or musculoskeletal disorders. The Emory Institutional Review Board approved the study, and all methods were carried out according to the approved protocol (IRB00082414).

### Experimental Set-up

Participants stood barefoot with their feet 22cm apart (approximately hip-width) on a custom movable platform (Factory Automation Systems, Atlanta, GA). Tape was placed around the participant’s feet to ensure consistent foot placement. All participants wore an overhead harness for safety and noise-canceling headphones playing white noise to eliminate auditory feedback. Under each foot was an independent force plate (AMTI, Watertown, MA, USA) that was embedded in the platform. Ground reaction forces from the force plates were collected at 1000 Hz. A 33-marker set based on a modified version of the Vicon Plug-in Gait model [22] that included additional foot markers (fifth metatarsal, medial and lateral heel, and medial malleolus) was used to track segmental movement.

### Balance Perturbations

**Figure 1:**
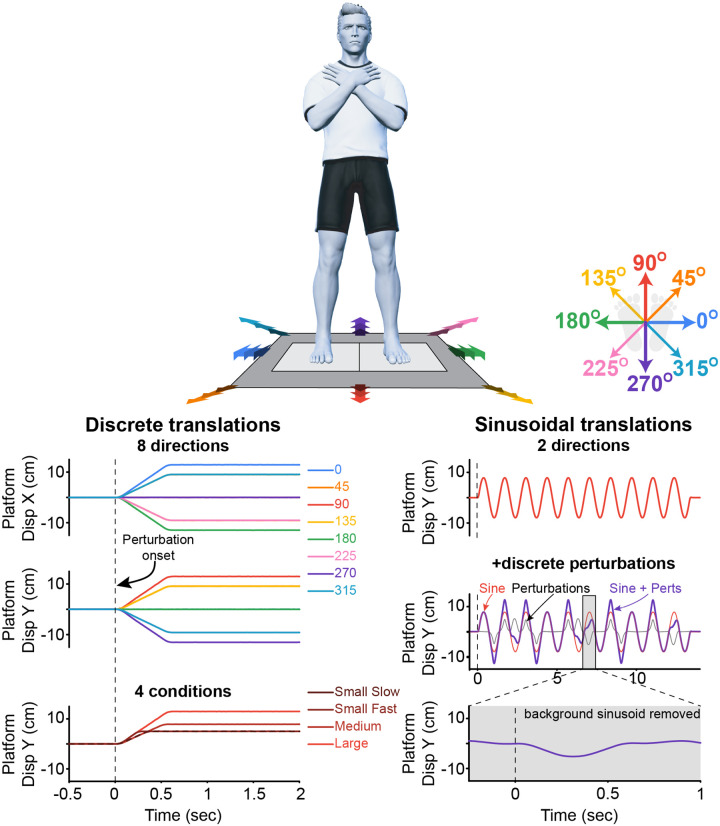
Experimental protocol. Participants were instructed to maintain a foot-in-place balance response while keeping their arms crossed on their chests. Participants experienced three different support surface translation conditions: discrete ramp-and-hold perturbations in 8 different directions and 4 different magnitudes, continuous sinusoidal movement in either the sagittal plane (moving along the 90° - 270° axis) or frontal plane (moving along the 0° - 180° axis), and a continuous sinusoidal movement with discrete perturbation superimposed. The superimposed perturbation was either in the same direction as the background sinusoid or against it (as illustrated in this figure). The vertical dashed line on all figures is the onset of the perturbation.

Participants were instructed to maintain standing balance in three different support surface translation conditions: discrete ramp-and-hold perturbations, continuous sinusoidal movement, and a continuous sinusoidal movement with discrete perturbation superimposed (Fig 1). The discrete ramp-and-hold perturbations occurred in 8 different directions: 0, 45, 90, 135, 180, 225, 270, and 315°, at 4 different magnitudes (displacement, velocity, acceleration): (1) 4.9 cm, 16 cm/s, 1.06 g (small slow); (2) 4.9 cm, 24 cm/s, 1.6 g (small fast); (3) 7.7 cm, 16 cm/s, 1.06 g (medium); (4) 12.6 cm, 24 cm/s, 1.6 g (large). Each perturbation was delivered 5 times in a randomized fashion, totaling 160 perturbations. The continuous sinusoidal movement occurred in 2 different directions: sagittal plane (moving along the 90° - 270° axis), and frontal plane (moving along the 0° - 180° axis), with a peak amplitude of 7.7 cm at 0.75 Hz. The continuous sinusoidal movement with discrete perturbations superimposed was the same as the sinusoidal movement but had the imposed perturbations that were either (1) 4.9 cm, 16 cm/s, 1.06 g; or (2) 4.9 cm, 24 cm/s, 1.6 g. The superimposed perturbations always occurred at the zero crossing, and were either in the same direction as the sinusoidal movement (accelerating) or opposing it (braking) (Fig 1). During all sinusoidal trials, 10 cycles were completed, for a trial duration of approximately 15 seconds. Each sinusoidal condition was delivered a total of 6 times, with it starting in either direction 3 times each, in a randomized fashion, totaling 36 perturbations. The ramp-and-hold perturbations were completed in one block, while all sinusoidal perturbations were completed in a second block. The order of these blocks was randomized across participants. To limit fatigue, rest breaks lasting at least 5 minutes were provided every 20 minutes. In all conditions, if the participant took a step, the trial was repeated once, and the stepping trial was excluded from further analysis. Stepping responses were identified in real-time as trials in which the magnitude of ground reaction forces for either leg dropped below 10 N.

### Data processing

Joint kinematics and kinetics were estimated from the measured limb segment marker data and ground reaction forces from both force plates. All marker data were filtered using a fourth-order low-pass filter with a 10 Hz cutoff, while ground reaction forces were filtered using a fourth-order low-pass filter with a 50 Hz cutoff. Inertial artifacts that occurred due to the movement of the platform were removed [30, 31]. Joint kinematics and joint torques were estimated using the inverse kinematics and inverse dynamics toolboxes in OpenSim (Gait 2892 model), respectively [32]. Prior to running these toolboxes, scaled subject-specific models were made using the Scale Tool. Horizontal CoM acceleration was calculated by dividing the ground reaction forces by the participant’s mass, and taken relative to the movement of the feet on the platform. CoM velocity and displacement were calculated as the weighted sum of all segmental masses from the kinematic data, and also taken relative to the movement of the feet on the platform. CoM displacement and velocity were up-sampled using linear interpolation to 1000 Hz for all further analysis.

To examine the balance-correcting response to the ramp-and-hold perturbations, all data were averaged across trials within each perturbation magnitude and direction for each participant and truncated to 0.25 seconds prior to the perturbation and 1 second after perturbation onset. Similarly, for the sinusoidal movements without perturbations, data were averaged across trials within each direction for each participant, and truncated to between 0.25 seconds prior to and 15 seconds after platform onset. In sinusoids with perturbations, balance-correcting responses to the superimposed perturbations were found by first subtracting the average response for the sinusoidal movements without perturbations (e.g., in Fig 1, subtracting the red trace from the purple trace to get the grey trace). Trials were then segmented and aligned to the onset of each perturbation based on the direction of the background sinusoid and the direction of the perturbation (e.g., the sinusoid and perturbation moving in the same direction versus the sinusoid and perturbation moving in opposite directions). Once all signals within the same condition were aligned to the onset of the perturbation, they were averaged and truncated to 0.25 seconds prior to the perturbation and 1 second after perturbation onset (Fig 1).

### Generalization of the delayed center of mass feedback model

**Figure 2:**
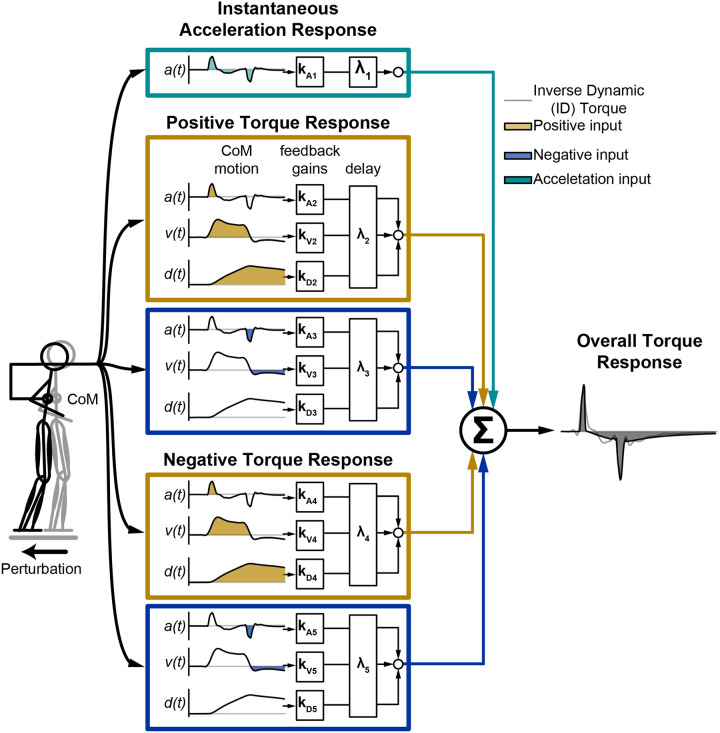
The delayed center of mass (CoM) model used to estimate the reactive joint torques. The delayed CoM model estimated torque as the quasi-linear sum of CoM deviation (acceleration, a; velocity, v; and displacement, d). The parallel CoM models (e.g., loops) enabled us to predict the positive and negative components of the torque response as well as the torque response to the positive and negative components of the CoM input. Adapted from [20]

To test our hypothesis that a delayed CoM feedback model of reactive balance joint torque [20] generalizes across different conditions, we tuned the gains and delays within each loop to optimize the fit between the ID-derived and the CoM feedback-derived joint torques for each participant, perturbation magnitude, and joint during the ramp-and-hold perturbations during the perturbations in the four cardinal directions (0, 90, 180, and 270°; “train”). All optimizations were performed using MATLAB R2022a (Mathworks, Natick, MA). Briefly, for the forward/backward perturbations (90 and 270°), CoM deviation in the sagittal plane was the input into the model, while CoM deviation in the frontal plane was the input to the right/left perturbations (0 and 180°). The mean background torque 0.25 seconds prior to the perturbation was calculated and removed from the overall torque response prior to model fitting. In prior work, we found a clear, instantaneous response to both the acceleration and deceleration of the CoM, particularly at the hip and knee [20, 29]. Thus, we fit a loop that only consisted of an acceleration gain and delay (*k*_*a*_ and *λ*; Fig 2). If this loop accounted for more than 95% of the variability in the ID-derived joint torque, the fitting procedure was ended. If this loop accounted for less than 95% of the variability, we fit the residual torque, as previously done [20, 29]: for the two loops reconstructing either the positive or negative torque response, optimization was performed to identify *k*_*di*_, *k*_*vi*_, *k*_*ai,*_ and *λ*_*i*_, where *i* indicates the *i*th loop. To prevent the algorithm from searching outside a physiologically relevant space and prevent model loops from reconstructing the same features within the response, we placed bounds on the gains and delays. During the fitting procedure, the fit of each loop was evaluated, and hand-tuning of the bounds was used if the loop poorly fit the data. For each joint, a total of 18 independent parameters could be fit. We have previously demonstrated that this method finds the optimal solution [20]. The gains and delays from these fits were then used for all generalizations.

We used the gains and delays from the cardinal directions (0, 90, 180, 270°, “train”) to predict the torque response in the diagonal directions (45, 135, 225, 315°, “test”). The X and Y gains were from perturbations within the same quadrant. For example, for a perturbation at 135°, the X and Y gains were the 180° and 90° gains, respectively. This was done to account for asymmetry in the response between leftward and rightward and forward and backward perturbation (e.g., in one direction, the leg is abducting while in the other, it is adducting). In this case, only gains from the same perturbation magnitude were used.

We next evaluated the ability of the CoM feedback model to generalize across perturbation magnitudes during the ramp-and-hold support surface perturbations. We used the gains and delays from each perturbation magnitude to predict the torque response to all other perturbation magnitudes. This was only done for the perturbations in the cardinal directions, and gains were matched to the perturbation direction (e.g., the small slow gains at 90° were used to predict the small fast, medium, and large torque responses to perturbations at 90°).

We used the gains and delays from each perturbation magnitude during the ramp-and-hold perturbations to predict the torque response to the sinusoidal movements. The gains were matched to the perturbation direction (e.g., the small slow gains at 90° were used to predict the response to the sinusoidal movement at 90°).

Lastly, we used the gains and delays from each perturbation magnitude during the ramp-and-hold perturbations to predict the torque response to the perturbation superimposed on the sinusoidal movement. The gains were matched to the perturbation direction, regardless of the direction of the background sinusoid (e.g., gains at 90° were used to predict the response to the perturbations in the 90° direction).

Generalization was assessed for ankle plantarflexion/dorsiflexion, knee flexion/extension, hip flexion/extension, and hip ab/adduction. Trials where the torque range was less than 10 Nm were excluded from further analysis due to a low signal-to-noise ratio. Note that we evaluated different torque cutoff values (other than 10 Nm); changing this did not significantly influence our results. Model accuracy in all cases was assessed using R^2^ and root mean square error (RMSE) with respect to the ground-truth biological joint torque. The RMSE was normalized by body weight for comparison to prior literature. The fit was considered a “good fit” if the average R^2^ was above 0.7 and the average RMSE was below 0.2 Nm/kg. These values are comparable to the performance of the current best-in-class machine learning algorithm for task-agnostic biological joint torque prediction for hip flexion and knee flexion [33]. We performed all analyses in MATLAB R2022a. Metrics are reported as the mean ± standard deviation unless otherwise noted.

## Results

### Multidirectional Generalization

**Figure 3:**
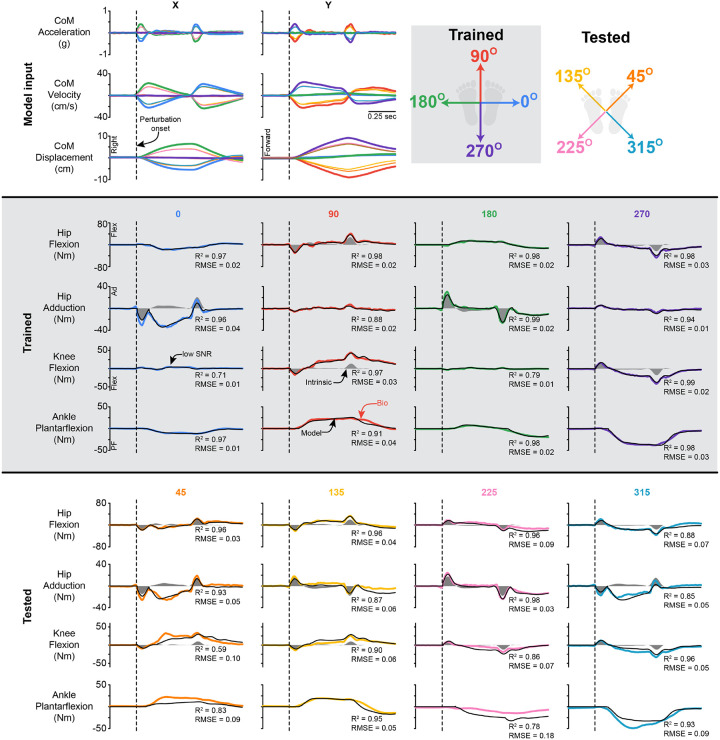
Direction Generalization representative traces. Representative fits for the CoM feedback model for perturbations in the trained directions (shaded - 0, 90, 180, and 270°) and tested directions (45, 135, 225, and 315°). The dashed line indicates the start of the perturbation. PF = plantarflexion, Flex = flexion, AD = adduction. Data presented are for the left leg. The black line is CoM feedback model estimate, while the colored lines are the inverse dynamic-derived joint torques. The shaded region is the instantaneous acceleration response. Note that the directions indicate the direction of platform movement, not CoM movement.

The delayed CoM feedback model, fit to joint torques during perturbations in the cardinal directions, was able to reconstruct joint torques during perturbations in the diagonal directions at the ankle, knee, and hip flexion and hip adduction (Fig 3). The model estimated hip, knee, and ankle torques in the training directions with a high R^2^ and low RMSE across all joints and directions (Table 1; Supplemental Fig 1). Representative time series are shown in Fig 3. Qualitatively, when looking across all joints and perturbation magnitudes, our model yielded a similar quality of fits across all magnitudes. While the model fit better in the training directions, the model estimated hip, knee, and ankle torques in the test directions with a high R^2^ and low RMSE across all joints and directions (Table 1; Supplemental Fig 1). It should be noted that while the R^2^ went down at all joints, the RMSE remained similar at the knee and hip, with a slight increase at the ankle. This is likely due to directions where the joint torque was low (e.g., a low signal-to-noise ratio); thus, the shape fitting was not as good, but the RMSE remained low.

Although the base structure of the delayed CoM feedback model had 18 independent parameters, the optimal fits at each joint required far fewer parameters. The model contained 18 parameters, allowing a single model to predict the response in all directions. For example, at the ankle, there was only a positive torque response for the perturbation at 90°, and only a negative torque response for the perturbation at 270°. Thus, the gains for the loops that fit the negative and positive components, respectively, were zero.

**Figure 4:**
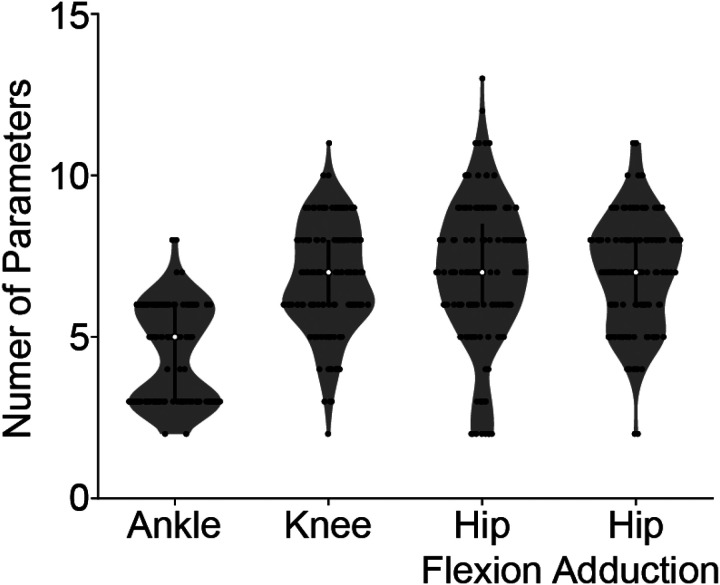
Number of parameters. While the base structure of the CoM model had 18 independent parameters, the optimal fits at each joint in the cardinal directions required far fewer parameters. The overall model structure, where parameters are optimized to zero during fitting, was employed to ensure that a single model structure could fit the torque response in all directions.

Across participants, on average 5 ± 2 parameters were needed to predict the ankle plantarflexion response, and 7 ± 2 for the knee flexion, hip flexion, and hip adduction response (Fig. 4). The number of parameters needed was similar across perturbation magnitudes (Supplemental Fig 2).

### Magnitude Generalization

**Figure 5:**
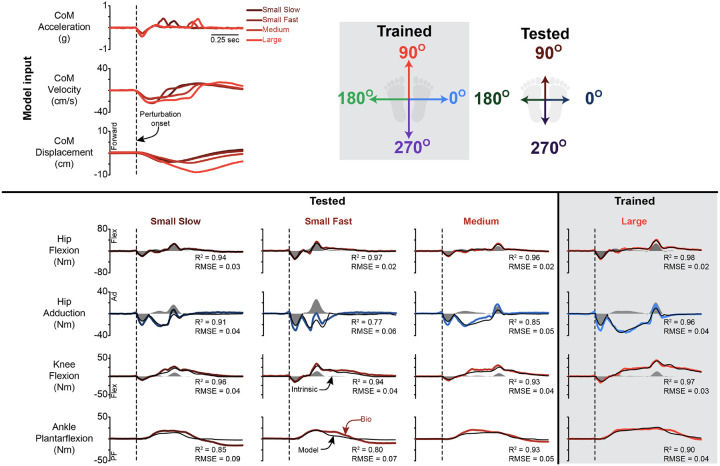
Magnitude generalization representative traces. Representative fits for the CoM feedback model for perturbations at one magnitude (Large–shaded) and tested at the three other magnitudes for perturbations at 90° for ankle, knee, and hip flexion, and 0° for hip adduction. The dashed line indicates the start of the perturbation. PF = plantarflexion, Flex = flexion, Ad = adduction. Data presented are for the left leg. The black line is CoM feedback model estimate, while the colored lines are the inverse dynamic-derived joint torques. The shaded region is the instantaneous acceleration response. Note that the directions indicate the direction of platform movement, not CoM movement.

The delayed CoM feedback model, which was fit at one perturbation magnitude, was able to generalize to other perturbation magnitudes at the ankle, knee, hip flexion, and hip adduction (Fig 5). The model estimated hip, knee, and ankle torques at all magnitudes with a high R^2^ and low RMSE (Table 2, Supplemental Fig 3). Representative time series are shown in Fig 5. Across all joints, the model trained on the medium perturbation generalized the best to other perturbation magnitudes when examining both the R^2^ and RMSE.

### Generalization to sinusoidal movements

**Figure 6:**
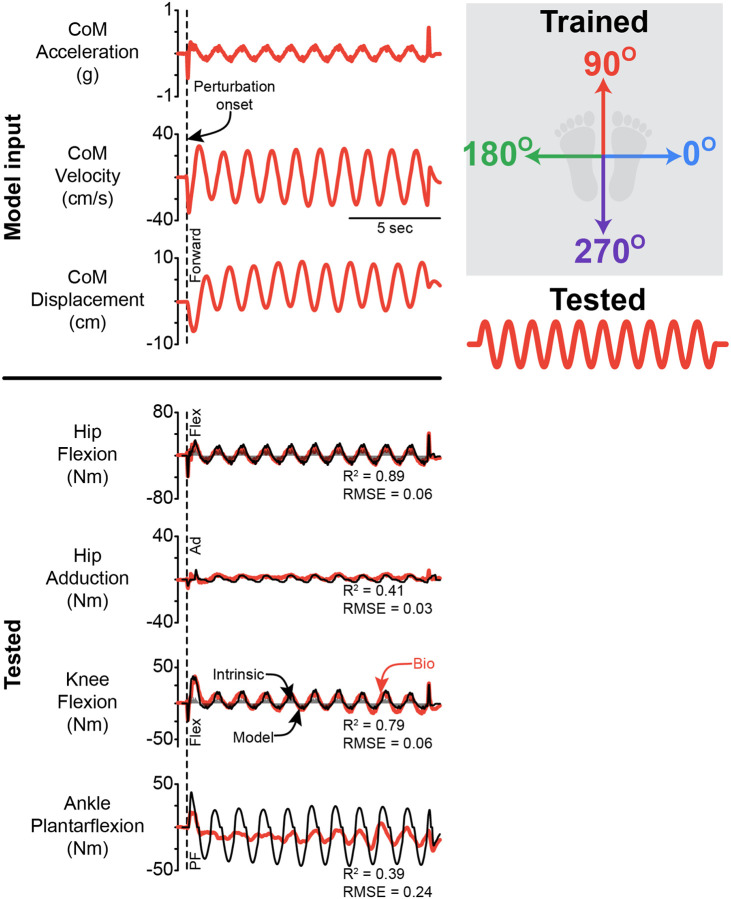
Representative traces of the generalization from ramp-and-hold perturbations to sinusoidal movements. Representative fits for the CoM feedback model that was trained on the medium ramp-and-hold perturbations, predicting the response to continuous sinusoidal movements. The dashed line indicates the start of the perturbation. PF = plantarflexion, Flex = flexion, Ad = adduction. Data presented are for the left leg. The black line is CoM feedback model estimate, while the colored lines are the inverse dynamic-derived joint torques. The shaded region is the instantaneous acceleration response.

The delayed CoM feedback model, which was fit to ramp-and-hold perturbations, was able to generalize to sinusoidal movements in hip flexion and marginally well at the knee, but not at the ankle or in hip adduction (Fig 6). The model estimated hip flexion torques at all magnitudes with a high R^2^ and low RMSE (Table 3, Supplemental Fig 4). The fit quality, as measured by both R^2^ and RMSE, decreased slightly for the estimation of knee torques (Table 3, Supplemental Fig 4). The model was unable to generalize at the ankle and hip adduction, with a low R^2^ and high RMSE (Table 3, Supplemental Fig 4). Representative time series are shown in Fig 6. For hip and knee flexion, the medium ramp-and-hold perturbation generalized to the sinusoidal movement the best, as indicated by both R^2^ and RMSE.

### Generalization to discrete perturbation superimposed on sinusoidal movements

**Figure 7:**
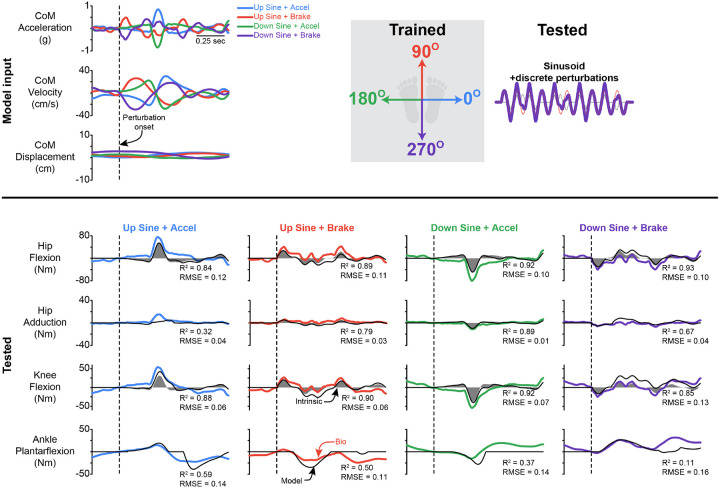
Representative traces of the generalization from ramp-and-hold perturbations to perturbations superimposed on the sinusoidal movements. Representative fits for the CoM feedback model that was trained on the small fast ramp-and-hold perturbations, predicting the response to the perturbation superimposed on the continuous sinusoidal movements. The dashed line indicates the start of the perturbation. PF = plantarflexion, Flex = flexion, Ad = adduction. Data presented are for the left leg. The black line is CoM feedback model estimate, while the colored lines are the inverse dynamic-derived joint torques. The shaded region is the instantaneous acceleration response.

The delayed CoM feedback model, which was fit to ramp-and-hold perturbations, was able to generalize to the discrete perturbations superimposed on sinusoidal movements for the hip flexion and knee flexion torque responses, but not at the ankle or for hip adduction torques (Fig 7). The model estimated knee and hip flexion torques for both slow and fast perturbations with a high R^2^ and low RMSE, while it was unable to generalize at the ankle and hip adduction, with a low R^2^, however, the RMSE remained relatively small (Table 4 & 5, Supplemental Fig 5). Representative time series are shown in Fig 7. There were no distinct differences in the quality of the fit for the slow versus fast perturbations. For hip flexion, the small-slow ramp-and-hold perturbation generalized to both the slow and fast superimposed perturbations the best when examining both the R^2^ and RMSE, while the large perturbation generalized the best at the knee to both the slow and fast superimposed perturbations.

## Discussion

In this work, we demonstrated the capability of a delayed CoM feedback model to estimate the biological multi-joint torque response to perturbations of varying direction and magnitude, as well as across movement contexts designed to capture the continuum from discrete to continuous balance challenges. Overall, the CoM feedback model generalized best across all conditions for hip flexion torque responses, closely followed by the knee flexion torque response. For the hip adduction and ankle plantarflexion torque response, the CoM feedback model generalized across perturbations of varying magnitude and direction, but was unable to generalize across movement contexts. The ability of the model to generalize across movement contexts for hip and knee flexion but not hip adduction and ankle plantarflexion may be related to the heavy reliance on the intrinsic mechanical response for hip and knee flexion, while the ankle relies more on neural feedback [20]. While prior work has demonstrated that delayed CoM feedback can predict the balance-correcting response at the ankle during walking [9], our results support prior findings that there are different feedback gains that modulate the balance-correcting responses at the ankle across different movement contexts [34]. Collectively, these results suggest that a single control vector (CoM position, velocity, and acceleration), can drive a hip or knee lower-limb wearable robot (i.e., exoskeleton, exosuit, or prosthesis) designed to restore, assist, or enhance balance in response to various perturbations and movement scenarios. Conversely, CoM kinematics could be used to control a multi-joint device that includes the ankle or frontal plane hip assistance to aid only in standing balance in response to various perturbations. Using a single control scheme to robustly predict the torque response at multiple joints (e.g., multiple actuators) can significantly simplify the control of lower-limb robotic exoskeletons designed to augment balance. As such, future work should explore methods for improving the generalizability across movement contexts at the ankle.

## Generalization

If implemented as a torque-control algorithm on lower-limb wearable robotic hardware (such as a powered exoskeleton, exosuit, or prosthesis), the assistive torque would closely mimic the biological response to balance perturbations with good temporal accuracy at all joints when responding to perturbations of varying magnitude and direction during standing. The delayed CoM feedback model that was trained on ramp-and-hold perturbations during standing generalized to ramp-and-hold perturbations at other directions and magnitudes for all joints. We observed a slight decrease in fit quality when the movement was not coplanar with the joint’s action (e.g., the 0° perturbation is orthogonal to hip flexion). The decrease in the fit quality is likely related to the small overall torque response, decreasing the signalto-noise ratio (Fig. 3). Despite the R^2^ decreasing in these conditions, the RMSE remained very low, indicating that the model continued to capture the small reactive torque response.

While the delayed CoM feedback model, trained on ramp-and-hold perturbations, generalized to sinusoidal movements as well as the perturbations superimposed on the sinusoidal movements for the hip flexion and knee flexion torque responses, it failed to do so for the hip adduction and ankle plantarflexion torque responses. The inability of the CoM feedback model to generalize across movement contexts for the ankle and the hip adduction torque response may be related to differences in the physiological pathways contributing to the torque response at each joint. A large portion of the hip flexion response is due to the feedforward intrinsic mechanical response [20]. Within our data, the feedforward intrinsic mechanical response accounted for, on average, 68% of the sagittal plane hip torque response across all magnitudes for perturbations in the sagittal plane (e.g., 90 and 270°; R^2^ = 0.68 ± 0.11; range = 0.20–0.98). In contrast, the ankle plantarflexion response relies entirely on neural feedback, with no feedforward intrinsic mechanical response [20]. The neural pathways mediating the response may differ between posture and movement, and throughout the movement [34]. More work is needed to determine how to improve the generalization across movement context of the CoM feedback model at the ankle and frontal plane of the hip.

The difference in reliance on feedforward versus feedback control at different joints has significant implications for the control of wearable robotic devices. The feedforward activation of muscles generates an instantaneous torque response at the time of the perturbation, with no delay [20]. Two limitations prevent the ability to actuate exoskeletons with a similar instantaneous response. First, all current balance augmenting exoskeletons require the perturbation to be detected, with the current fastest detection being approximately 150 ms [10]. Second, there is an additional delay based on the bandwidth of the device, which prevents actively controlled actuators on the exoskeleton from mimicking the instantaneous intrinsic biomechanical response. This results in open questions like: What are the consequences of failing to deliver the intrinsic mechanical response, considering both its role in enhancing the balance-correcting response and its impact on how individuals perceive the assistance? Second, prior work suggests that exoskeleton torque should be applied faster than the muscular response [12]. At the hip, due to the intrinsic mechanical response, this is not possible. While it would be possible when actuating the ankle, the CoM feedback model has multiple delays [20]: how much faster than the human response should we actuate, and is this consistent for each independent delay within the model? Future work is still required to answer these questions, as well as others, before exoskeletons can be widely deployed as balance aids.

## Comparison to other control methods

Our proposed CoM feedback controller does not require an explicit perturbation detector to actuate; it updates the applied torque based on continuous measures of the CoM. While we could have employed other methods, including alternative forms of CoM control, splines, or machine learning, these methods all require identification of the perturbation to apply the “assistive” torque [9–15]. Currently, the fastest detection is approximately 150 ms [10]. As stated previously, we want to actuate the device faster than the biological response at about 50 ms [12]. This currently cannot be achieved without the device having knowledge of when the perturbation occurs, which limits its ecological relevance. Since our proposed CoM feedback controller runs continuously without the need for a perturbation detector, we would be able to actuate artificially fast, only being limited by the bandwidth of the device.

Our CoM feedback controller requires very little training data to achieve the observed generalizability. An alternative approach for predicting the biological torque response would be to leverage recent advances in machine learning [33], although it has not been validated for predicting the biological joint torque response to perturbations, early work predicting other biological variables suggests that it is a viable solution [35]. While machine learning approaches have been shown to generalize to untrained conditions [33], to achieve comparable generalization to the CoM model, machine learning models require millions of labels from data sets with large heterogeneity [36]. In comparison, participants only needed to complete approximately 20 trials, with each trial lasting less than 5 seconds—for a total of less than 2 minutes of data collection and approximately 80,000 labels. Moreover, although the tasks are different, we achieved comparable R^2^ and RMSE values with the current best-in-class generalizable machine learning model [33]. Lastly, we demonstrate that the CoM feedback model can generalize across movement context, generalizing from the ramp-and-hold perturbations to the sinusoidal movement; it is highly unlikely that a machine learning algorithm could achieve similar generalization unless the training task set included conditions similar to the test conditions.

The CoM feedback controller required relatively few parameters to be optimized to capture the complexity of the reactive torque response. Spline-based control with up to 10 optimized parameters has been quite effective in minimizing the metabolic cost of walking [5, 37]. Our CoM feedback controller required a similar number of parameters to be optimized as spline-based control; however, it achieved task-agonistic generalization without the need for a lookup table. One major limitation of splines for balance augmentation is that individual spline fits would be required for each possible perturbation direction, magnitude, and movement context, resulting in an unrealistic combination of parameters, preventing translation outside the laboratory.

## Limitations

One limitation of the current study is that, although we provided sinusoidal movement as an approximation of walking, it does not fully emulate motion during walking. Due to experimental limitations, we were unable to provide perturbations during walking. Future work should investigate whether the delayed CoM feedback model from standing balance can be generalized to walking balance. Second, while we explored generalization, all the perturbations were only support surface perturbations. It is unclear whether this model generalizes to other types of perturbations (e.g., rotations, center of mass perturbations, visual, internal, etc.). Another limitation of the current work is that the largest perturbation that was applied was 12.6 cm. This was due to experimental constraints on the maximum displacement of the platform. This perturbation size poses a small to moderate challenge for healthy young adults. While stepping reactions were observed, all participants were able to complete all perturbation conditions with a feet-in-place response. The CoM feedback model should be tested on larger perturbation magnitudes in different directions to examine the generalizability of this approach thoroughly. Lastly, while the hand-tuned fits were good for nearly all subjects, there were a few cases when this was not the case. Although this issue only occurred in low-torque conditions, it may be contributing to the lack of generalizability. We are currently evaluating ways to improve the generalizability and robustness of the model.

## Conclusion

In this work, we demonstrate that a delayed CoM feedback model can accurately predict the reactive torque response at the hip, knee, and ankle in response to balance perturbations in varying directions and magnitudes. Moreover, we found that this model can generalize across movement contexts at the hip and knee. This serves as an encouraging proof-of-concept, suggesting that a delayed CoM feedback model has the potential for real-time control of a lower-limb wearable robotic device for balance augmentation. Moreover, our approach may be able to improve balance in any legged system where CoM state can be estimated in real-time (e.g., bipedal robots or humanoids).

## Supplementary Files

This is a list of supplementary files associated with this preprint. Click to download.

- JNERJakubowski2026Supplemental.docx

## Figures and Tables

**Table 1:** Multidirectional Generalization

	*Ankle*		*Knee*		*Hip Flexion*		*Hip Adduction*	
	R^2^	RMSE (Nm/kg)	R^2^	RMSE (Nm/kg)	R^2^	RMSE (Nm/kg)	R^2^	RMSE (Nm/kg)
Trained	0.95 ± 0.04	0.03 ± 0.02	0.95 ± 0.06	0.03 ± 0.02	0.96 ± 0.05	0.03 ± 0.02	0.93 ± 0.09	0.03 ± 0.01
Tested	0.85 ± 0.15	0.08 ± 0.03	0.89 ± 0.09	0.07 ± 0.03	0.91 ± 0.08	0.04 ± 0.02	0.84 ± 0.14	0.04 ± 0.02

**Table 2:** Magnitude Generalization

	*Ankle*		*Knee*		*Hip Flexion*		*Hip Adduction*	
	R^2^	RMSE (Nm/kg)	R^2^	RMSE (Nm/kg)	R^2^	RMSE (Nm/kg)	R^2^	RMSE (Nm/kg)
Tested	0.92 ± 0.07	0.05 ± 0.03	0.94 ± 0.06	0.05 ± 0.04	0.92 ± 0.09	0.05 ± 0.03	0.89 ± 0.10	0.04 ± 0.02

**Table 3:** Sinusoid Generalization

	*Ankle*		*Knee*		*Hip Flexion*		*Hip Adduction*	
	R^2^	RMSE (Nm/kg)	R^2^	RMSE (Nm/kg)	R^2^	RMSE (Nm/kg)	R^2^	RMSE (Nm/kg)
Tested	0.24 ± 0.17	0.22 ± 0.10	0.70 ± 0.18	0.13 ± 0.04	0.77 ± 0.17	0.10 ± 0.04	0.20 ± 0.22	0.18 ± 0.07

**Table 4:** Sinusoid + Perturbations Generalization – Fast

	*Ankle*		*Knee*		*Hip Flexion*		*Hip Adduction*	
	R^2^	RMSE (Nm/kg)	R^2^	RMSE (Nm/kg)	R^2^	RMSE (Nm/kg)	R^2^	RMSE (Nm/kg)
Tested	0.50 ± 0.23	0.14 ± 0.06	0.84 ± 0.12	0.11 ± 0.05	0.88 ± 0.11	0.11 ± 0.04	0.56 ± 0.29	0.18 ± 0.06

**Table 5:** Sinusoid + Perturbations Generalization – Slow

	*Ankle*		*Knee*		*Hip Flexion*		*Hip Adduction*	
	R^2^	RMSE (Nm/kg)	R^2^	RMSE (Nm/kg)	R^2^	RMSE (Nm/kg)	R^2^	RMSE (Nm/kg)
Tested	0.61 ± 0.24	0.11 ± 0.04	0.85 ± 0.11	0.10 ± 0.05	0.87 ± 0.13	0.08 ± 0.04	0.54 ± 0.29	0.13 ± 0.05

## Data Availability

The data from the current study are available from the corresponding author upon reasonable request.

## Abbreviations

CoM: Center of Mass
PF: Plantarflexion
Flex: Flexion
AD: Adduction
SS: Small Slow
SF: Small Fast
M: Medium
L: Large
